# Management of Breathlessness in Palliative Care: Inhalers and Dyspnea—A Literature Review

**DOI:** 10.5041/RMMJ.10357

**Published:** 2019-01-28

**Authors:** Helen Senderovich, Akash Yendamuri

**Affiliations:** 1 Geriatrics & Pain Medicine & Palliative Care Physician, Baycrest Health Sciences, Toronto, ON, Canada; 2 Assistant Professor at the Department of Family and Community Medicine, Division of Palliative Care, University of Toronto, Toronto, ON, Canada; 3 Baycrest Health Sciences, Toronto, ON, Canada

**Keywords:** Dyspnea, inhalers, opioids, palliative care, shortness of breath

## Abstract

**Introduction:**

Dyspnea is prominently observed in palliative care (PC). Dyspnea can be multifactorial, primarily caused by obstructive or restrictive lung diseases or secondarily induced by various comorbidities. Numerous interventions exist, with route of administration and efficacy requiring further discussion. Despite opioids being the first line of treatment, their adverse effects lead to reluctance on the side of patients to take them, creating limitations in patient management planning.

**Objectives:**

This paper reviews and highlights the role of inhalers for dyspnea management in PC.

**Methods:**

The CINAHL, CENTRAL, and OVID databases were searched for scholarly articles on the role of inhalers in dyspnea management from 1998 to the present. A grey literature Internet search was also performed via Google, the World Health Organization, and CareSearch. Twenty-five articles relevant to the subject at hand were located and summarized. The Cochrane Systematic Reviews of Health Promotion and Public Health Interventions Handbook was consulted for structuring.

**Result:**

Isolated bronchodilators can be effective in dyspnea management. However, combination with opioids leads to a 52% reduction of dyspnea, demonstrating efficacy of their combined use. There is a role for conventional inhalers not only in patients afflicted with chronic obstructive pulmonary disease, but also in those where obstruction is reversible, and in cases of dyspnea not yet diagnosed.

**Conclusion:**

Inhalers can be utilized as adjuvant therapy to opioids, to limit opioid use, augment responses to dyspnea, and/or minimize opioid side effects, especially in opioid-naïve patients. Correct administration can increase the efficacy of short-acting beta-agonists, long-acting beta-agonists, short- and long-acting anticholinergic agents, and inhaled corticosteroids, achieving reduction and alleviation of dyspnea.

## INTRODUCTION

Dyspnea is a common symptom which manifests in palliative care (PC), with 70% of patients suffering from dyspnea during their last 6 weeks of life.^1^ It arises from a plethora of conditions, including malignancy, treatment-related causes (e.g. lobectomy, pneumonitis, medications causing fluid retention, or bronchospasm), infection, anemia, end-stages of cardiac, pulmonary, kidney, and liver illnesses, as well as psychological factors such as increased anxiety.^2^

At present, a wide array of treatment modalities is used to alleviate dyspnea in PC. These include opioids, anxiolytics (represented by benzodiazepines and neuroleptics), diuretics, and non-pharmacological interventions (e.g. the use of a fan, oxygen, positioning, physiotherapy, and occupational therapy). Adding further to the scope of treatments available, there are different routes of administering medications (i.e. oral, parenteral, and nebulized). However, despite these approaches to alleviate dyspnea, there are numerous side effects and limitations in their delivery.

Palliative care is the spectrum of medicine that focuses on the quality of life for patients afflicted with severe illnesses. It is directed to address the physical, social, psychosocial, and spiritual domains of care. Interchangeable words used are “end-of-life care” or “comfort care,” for example. The management goal is to provide patients with comfort and dignity up until the end of life, while respecting their wishes and acting in each patient’s best interests, adhering to culture, tradition, and values. In addition to using the least aggressive intervention possible, a further goal is to control the symptoms and minimize the side effects in this frail population with multiple comorbidities. Pharmaceuticals can be extremely harmful to patients, so physicians must first consider whether an alternative treatment or drug is clinically appropriate. The benefits to prescribing narcotics and controlled substances must be weighed against their potential risks when used in the long term.^3^ Conventional treatment modalities which may work for a relatively healthier patient population could have devastating effects on frail and vulnerable PC patients. As such, the risks and benefits must be assessed, and alternatives and/or adjuvant therapy should be sought.

The objective of this review was to highlight the role of inhalers in dyspnea management in PC.

## METHODS

The CINAHL, CENTRAL, and Ovid (MEDLINE, PsychINFO, Embase) databases were searched for recent scholarly articles on the role of inhalers in dyspnea management from 1998 to the present. A grey literature search was also conducted using the same search strategies, and included Internet searches of Google, the World Health Organization, and CareSearch. Although 40 articles were identified, only 25 were deemed relevant to the subject at hand. These included evidence-based cases and literature reviews, prospective and retrospective studies, guidelines, and randomized control trials (RCT); only one paper reported directly on a RCT, while the others quoted RCTs as sources of information. These articles were critically analyzed and summarized. Corroborative themes were identified, and the authors responsible for the contributing research were cited as they came up. Although this article is not a systematic review, the Cochrane Systematic Reviews of Health Promotion and Public Health Interventions Handbook was also consulted for appropriate definitions, number of reviewers, and transparency of information. To further emphasize opioids use and their nature, a theoretical case study was applied to this literature review with relevant points being associated as they came up.

## THEORETICAL CASE STUDY

Mrs X, a 76-year-old female of African descent with a history of end-stage lymphoma, complicated by pleural effusion, anemia, and cachexia, was admitted to a PC unit. She was a non-smoker. Soon after admission, her dyspnea became more pronounced, but it was managed with a short-acting beta-agonist (SABA). Salbutamol, two puffs every 4 hours, was initiated by the covering physician as the patient refused opioids due to associated sedation with its use. The patient responded partially to the above intervention and became comfortable at rest, but still demonstrated signs of dyspnea with minimal mobility and/or conversation. An inhaled corticosteroid (IC) (fluticasone) was added to the medication regimen, 500 μg every 12 hours. The patient’s condition stabilized and her dyspnea became well controlled. After 2 days, the senior physician, returning to duty, discontinued inhalers due to the observed improvement in the patient’s condition and because of the documented evidence indicating that opioids were the treatment of choice for dyspnea in terminally ill patients. However, within 3 days post-discontinuation of inhalers, staff noted an increase in shortness of breath which correlated with an escalation of anxiety. Despite the patient’s initial apprehension with regard to opioid use, this time she consented to the use of opioids, which were initiated on an as-needed basis (PRN). Hydromorphone 0.25 mg orally once or twice daily over the next 5 days was required. Despite initiation of opioids, she continued to have dyspnea, and the dosage was increased to four times a day, with the patient’s consent. Nevertheless, her dyspnea continued to escalate and additional opioids were utilized PRN. She rapidly declined and died the following day.

## DISCUSSION

The first line of treatment for dyspnea is opioids, but this is not without limitation, as they can have detrimental side effects due to well-known complications; other options should therefore be considered. In addition, when made aware of the potential side effects, patients may exercise their right to refuse this treatment. This should prompt other means of alleviation, and inhalers such as SABA/ long-acting beta-agonists (LABA), anticholinergics, and IC may be the options. Our theoretical case study exemplifies situations where rapid titration of opioids was not the best alternative.

### Isolated Bronchodilator Use

In advanced pulmonary disease, there are many options to manage dyspnea. Bronchodilators, including anticholinergics and SABA, are used to treat reversible bronchospasm.^4^ Albuterol (SABA) has been used as a rescue inhaler due to its rapid onset of action and was found to improve dyspnea related to exertion in patients with chronic obstructive pulmonary disease (COPD) in a RCT.^5^ Salmeterol xinafoate and salbutamol/clenbuterol (both LABAs) have proven to be even more efficacious in comparison.^5^ Beta-agonists also improve mucociliary clearance, where the lack of clearance can contribute further to manifestations of dyspnea. Hence, their use is more beneficial when used before chest therapy.^6^ The risks associated with beta-agonist use include tremors, anxiety, hypokalemia, and reflex tachycardia. Tiotropium bromide is an anticholinergic drug which has been demonstrated to be beneficial for patients with COPD by decreasing dyspnea and frequency of exacerbations, improving lung function, and reducing dynamic hyperinflation. A greater than 10% vital capacity improvement has also been noted.^5^ Beta-agonists and anticholinergics have contrasting effects. Anticholinergics were shown to reduce the number of dyspneic episodes, maintained longer bronchodilating effects at the same level (up to 85 days), and resulted in higher levels of forced expiratory volume (FEV1) compared to beta-agonists.^5^ Furthermore, there was a 17% reduction in COPD exacerbations when comparing tiotropium to salmeterol. Comparing tiotropium and indacaterol, similar results were found with respect to FEV1 and dyspnea.^7^ Inhaled corticosteroids have been proven to be very effective in the treatment of dyspnea for specific diagnoses. In addition to COPD, airway obstruction induced by cancer is another condition where IC were beneficial. One paper reported a combined obstructive/restrictive pattern in 47% of cancer patients, and an isolated restrictive pattern in 41%.^4^ Bronchospasm is another cause for dyspnea in advanced cancer patients. One study revealed that 52% of patients with an obstructive respiratory pattern had benefited from maximal bronchodilation—and Mrs X could exemplify this type of patient, where discontinuation of inhalers possibly led to escalation of dyspnea.^8^ Other conditions for which the use of IC is advocated include: pulmonary inflammatory conditions, lymphangiosis carcinomatosa, radiation pneumonitis, and superior vena cava syndrome.^9^ Combination therapy of IC with LABAs has proved to be another efficacious way to alleviate dyspnea; noticeable improvements have been seen in those suffering from COPD and asthma on such a regimen.^10^ Despite the merits of IC, there are risks associated with their long-term use. These include muscle weakness, chest wall and diaphragm weakness, oral thrush, and hyperglycemia.^10^

### Bronchodilators and Opioids—Combination Therapy

Inhalers used in combination with opioids produced significant reductions in dyspnea compared to isolated use. This was supported by a comparative study.^11^ Acute dyspnea was diagnosed in 116 PC patients within a 2-year period. These patients were divided into five groups: Group 1 received morphine and oxygen; Group 2 received morphine, bronchodilators, and oxygen; Group 3 received bronchodilators and oxygen; Group 4 received oxygen; and Group 5 received no medical treatment. The results showed that Group 2 had a 52% success rate in alleviating dyspnea compared to only 22% of the group treated with Group 3. However, the group treated with morphine and oxygen (Group 1) had a 67% success rate in dyspnea alleviation, with an average of 41% of patients that were adequately treated. Statistically significant differences were found (*P*<0.001) between Groups 1 and 2 compared to Groups 3 and 4.^11^ One article supported the idea of combination therapy, stating that there was an increased risk of cardiovascular events in those not combining an opioid with a non-opioid intervention.^12^

### Nebulized Furosemide

There are other inhalants that are used and that have shown modest benefits, one of which is nebulized furosemide. A small number of studies found relief of dyspnea for 4 hours within 20–30 minutes following administration of 20 mg of nebulized furosemide. This reduced the respiratory rate (RR) and accessory muscle use in patients refractory to opioid treatment.^13^ Another study reported reductions in dyspnea, cough, and tachypnea with the same dosage of nebulized furosemide delivered 4 times a day in PC patients.^10^,^14^ In comparison, nebulized morphine treatment yielded ambiguous results, and the authors of one review recommended that decisions for nebulized morphine treatment should be made on a case-by-case basis.^15^

### The Role of Benzodiazepines

In the event that opioids do not provide adequate relief of dyspnea, benzodiazepines are used as a second-line treatment.^16^ When breathlessness occurs, the ability to differentiate between true dyspnea and anxiety becomes difficult as both symptoms contribute to each other, escalating the problem. It is believed that the continuous sensation of dyspnea experienced by the patient is due to the lack of anxiolytic targeting by initial management. Midazolam, sublingual lorazepam, or, rarely, diazepam is used; however, their effectiveness with isolated use compared to their combination with other treatment modalities is still unclear.^4^,^17^ In patients with advanced cancer and COPD, there was no statistically significant difference between the use of benzodiazepine and morphine, although drowsiness and somnolence were less prominent in patients receiving benzodiazepine versus morphine.^16^

### Opioids

A widely used approach to relieving dyspnea in PC patients was the use of opioids, with morphine being the mainstay drug. The side effects of opioid use have remained significant, regardless of dyspnea improvement, due to frequently observed respiratory depression and constipation. Sedation has previously been considered worrisome and remains a concern for a significant number of patients and their families, although it was found that most patients become pharmacologically tolerant to the effects of opioids within 1–2 weeks.^4^ This apprehension to sedation and subsequent respiratory depression was apparent with Mrs X from our theoretical case study. Nevertheless, there are still some positive aspects to opioid use other than dyspnea alleviation. Lower hospital admission rates for congestive heart failure were found in new opioid users in patients with COPD in the community setting, with no additional cardiac-related mortality causes.^12^

While there are no efficacious differences between parenteral and oral use of opioids, the latter is preferred.^17^ The reason is that it is the least invasive, most convenient route of administration, and is cheaper compared to other forms of administration.^18^,^19^ Another study also revealed that nebulized opioids demonstrated no statistical significance.^17^ This was confirmed by a systematic review,^20^ which revealed that systemic opioids provide greater relief of dyspnea compared to nebulizers with regard to refractory breathlessness in COPD patients using a visual analog scale, a chronic respiratory questionnaire, a 6-minute walk test, and the oxygen cost diagram. Even when heterogeneity was removed, the systemic opioid remained superior to nebulized ones. This was corroborated with another study involving a 10-year-old boy with end-stage cystic fibrosis. The patient was given incremental dosages of nebulized morphine, starting with 2.5 mg and increasing to 12.5 mg. No significant differences were found with differing dosages, apart from a 9 mmHg increase in venous carbon dioxide levels when the 12.5 mg dosage was given.^21^ However, in one case report a 74-year-old woman with a history of hypertension was admitted as a result of her complaints (weight loss, pain, and decreased appetite), and investigations revealed that she had metastatic disease.^22^ She was initially prescribed 10 mg of oral continuous release morphine three times a day for pain control. She then began to complain of shortness of breath. To deal with the dyspnea, 4 mg of nebulized morphine was prescribed. Respiratory depression ensued 15 minutes post-administration, with a RR of 4–5 breaths per minute, hypotension (blood pressure of 70/40 mmHg), and partial response to command. This prompted the use of intubation and resuscitation.^22^

The merits of opioid use to help with dyspnea are apparent, and it is a first-line treatment, but the side effects of opioid use are considerable. This may relate to our hypothetical case study with Mrs X where rapid titration of opioids in an opioid-naïve and cachexic patient might have led to rapid decline and an earlier-than-expected terminal outcome. Dosage plays a pivotal part in providing relief and minimizing side effects of respiratory depression. A RCT with 83 participants highlighted that 10 mg of sustained-release oral morphine can yield positive outcomes when given to responders, resulting in a 62% reduction in dyspnea, a number needed to treat (NNT) of 1.6, and a *P* value of <0.001.^23^

### Inadequate Management

One of the main problems with using inhalers, leading to suboptimal levels of relief, is the lack of knowledge pertaining to correct delivery method. Whether the primary or ancillary level of care, standards of methodology should be established to ensure beneficial results. When providing care, only 34.9% of hospice nurses performed the correct steps when administering a dry powder inhaler (DPI), 38% with nebulizer use, 44.7% with a metered-dose inhaler (MDI), spacer, and mask, 49.9% with a MDI and spacer, and 67.6% correctly delivering MDI alone. This demonstrated that there was a noticeable knowledge gap in techniques to properly deliver and use inhalers. There are some noticeable factors associated with these statistics leading to undesirable delivery techniques contributing to the overall results above. Those who had been nurses for more than 11 years only performed 2.61 of the 10 (*P*<0.05) steps correctly when demonstrating appropriate DPI technique compared to those with less than 10 years of experience (6.40/10). Factors associated with subpar nebulizer techniques include lack of hospice certification (4.6 steps correctly performed out of 14; *P*<0.05) and being uncomfortable using a nebulizer (2.20/14; *P*<0.05). Being uncomfortable with MDI technique was also seen, with 3.14 steps correctly performed out of 8 (*P*<0.05). Just 2.96 steps of the 7 were performed correctly (*P*<0.05), with nurses not checking patient inhaler techniques with MDI and a spacer. Upon further investigation, hospice nurses who participated in this endeavor attributed lack of training, specifically with the use of DPIs, years in hospice care, and lack of personal use to the suboptimal execution of inhaled drug delivery, which consequently leads to inadequate dyspnea relief for patients noted above.^24^ Metered-dose inhalers are the preferred route due to improved compliance, simplification of therapy, reduced medication usage, and lowered costs. When delivered using the correct method, MDIs have the same effect as using a nebulizer.^7^ Correct inhaler technique is crucial for the alleviation of patients’ dyspnea, and nurses’ ability to educate patients in this is vital.

### Knowledge Translation

Proper education of medical practitioners on appropriate inhaler use and the correct use of dry-powder inhalers and metered-dose inhalers is crucial and suggested to ensure adequate response to inhalers and symptom relief. Therefore the right knowledge can be passed on to patients to increase the benefit of the treatment and avoid suboptimal drug delivery and, as a result, subpar symptom control.

The use of conventional inhalers and nebulizers is recommended for patients afflicted with COPD, or if obstruction (possibly reversible) is an issue. A SABA could be added if there is wheezing. Even without wheezing, a SABA could be beneficial, and this is what was noted in Mrs X.^25^ If there is no response with a therapeutic trial of inhalers, this therapy should be stopped.^4^ A SABA should also be considered for patients with dyspnea not yet diagnosed as some may have a history of pulmonary illness and have never seen a physician or been officially diagnosed.

There should also be awareness and an understanding of the important roles that SABA, LABA, short- and long-acting anticholinergic agents, and IC have. Their use is not limited to patients suffering with COPD; they could also be used in those with end-stage chronic illnesses and cancer when dyspnea caused by bronchospasm is a concern. In addition, combined usages of the above-indicated inhalers and opioids may help minimize side effects related to opioid use, achieve symptom control, and improve a patient’s quality of life.

## CONCLUSION

In summary, there are clearly a plethora of management options to alleviate dyspnea in PC patients. Patients afflicted with dyspnea may hesitate to use opioids in mild to moderate cases. Although sedation is a side effect that decreases in prevalence as time progresses, respiratory depression remains a strong manifestation with severe consequences. The current practice to focus on opioids as the treatment of choice in terminally ill patients with dyspnea is a significant barrier to combination therapy or isolated inhaler usage without opioids. Nevertheless, these alternatives to opioids alone should be considered for management of mild and moderate cases of dyspnea, particularly if patients have responded well to such therapies in the past. Broadening the scope of relief, inhalers including SABAs, LABAs, anticholinergics, and/or IC could be useful, as the risks of side effects are minimal. The role that inhalers play is invaluable, and physicians must not be too hasty to dismiss inhalers for opioid use in responders, or if symptoms can be controlled just with inhalers. Physicians should be mindful that an inhaler could be an option in patients who have dyspnea not yet diagnosed, suspected bronchospasm of multifactorial origin, and in those who never sought prior medical attention and who responded well to trial use of inhalers. The culmination of these endeavors will lead to diagnostic advances and therapeutic alternatives to effectively manage patients afflicted with dyspnea while respecting their values, beliefs, and minimizing opioid-related side effects.

## LIMITATIONS

The limitations of this review relate, in general, to the content of the available literature. While the literature is plentiful on opioids, SABAs, and LABAs, there is minimal information available regarding drug classes less often used in dealing with dyspnea in PC, e.g. benzodiazepines, furosemide, and combination therapies with the above medications. Hence, this review could only relate to the few recent papers on these latter treatment modalities. Small sample sizes and wide confidence intervals could imply inaccuracies—sample size in certain studies was small with confidence intervals that were too broad to extract a reliable conclusion.^16^ Nevertheless, 95% confidence intervals were the standard.^7^,^16^ Certain articles only provided *P* values for a handful of their findings, making it difficult to find a valid application of the study to current practice.^24^ A few articles reported on initial studies, such as one with no other retrospective studies available for comparison.^11^ Other sources provided statistical information, but the validity and reliability of these statistics were not clear.^1^,^5^–^7^,^10^,^13^,^14^ The number of existent RCTs was minimal, and the blinding properties of the study design were also not clear.^11^ In addition, loss to follow-up and the short duration of some studies may have impacted the reported outcome.^8^,^23^ The selected papers covered a wide range—from retrospective studies to highly opinionated pieces—which could lead to information bias.

## FUTURE RESEARCH

Additional emphasis should be placed on other approaches to alleviate dyspnea with higher-powered research, such as RCTs with longer follow-up, larger sample sizes, and with standardized inclusion and exclusion criteria to minimize the bias in the reported outcome. Certain conclusions drawn in this paper were based on minimal sources, such as delivery method efficacy, which requires further research. Future studies should focus on the combined use of SABA, LABA, IC, anticholinergics, and opioids on various patient demographics, especially in PC settings. In addition, the use of nebulized furosemide in isolation and in combination with SABA, LABA, anticholinergics, IC, and opioids should be further researched. The use of benzodiazepines in the treatment of dyspnea should also be looked into further, either alone or in combination with other inhalers. This is especially important when anxiety is a concern, to determine its effectiveness and better evaluate the side effect associated risks versus its benefits.

## Abbreviations

COPD: chronic obstructive pulmonary disease
DPI: dry powder inhaler
FEV1: forced expiratory volume
IC: inhaled corticosteroids
LABA: long-acting beta-agonist
NNT: number needed to treat
PC: palliative care
PO: orally
PRN: as needed
RCT: randomized control trials
RR: respiratory rate
SABA: short-acting beta-agonist.

## References

[1] Dudgeon DJ (2002). Managing dyspnea and cough. Hematol Oncol Clin North Am.

[2] Back I (2016). Dyspnoea/breathlessness. Palliative Care Guidelines Plus website.

[3] The College of Physicians and Surgeons of Ontario Policy Statement #7-16: Prescribing Drugs.

[4] Thomas JR, Von Gunten CG (2003). Management of dyspnea. J Support Oncol.

[5] Runo JR, Ely EW (2001). Treating dyspnea in a patient with advanced chronic obstructive pulmonary disease. West J Med.

[6] Janssens JP, de Muralt B, Titelion V (2000). Management of dyspnea in severe chronic obstructive pulmonary disease. J Pain Symptom Manage.

[7] Ferguson GT, Make B (2012). Management of stable chronic obstructive pulmonary disease. UpToDate.

[8] Dudgeon DJ, Lertzman M (1998). Dyspnea in the advanced cancer patient. J Pain Symptom Manage.

[9] Kloke M, Cherny N, ESMO Guidelines Committee (2015). Treatment of dyspnoea in advanced cancer patients: ESMO Clinical Practice Guidelines. Ann Oncol.

[10] Indelicato RA (2006). The advanced practice nurse’s role in palliative care and the management of dyspnea. Topics in Advanced Practice Nursing eJournal.

[11] Wiese CH, Barrels UE, Graf BM, Hanekop GG (2009). Out-of-hospital opioid therapy of palliative care patients with “acute dyspnoea”: a retrospective multicenter investigation. J Opioid Manag.

[12] Rocker G, Bourbeau J, Downar J (2018). The new “opioid crisis”: scientific bias, media attention, and potential harms for patients with refractory dyspnea. J Palliat Med.

[13] Owens D (2009). Nebulized furosemide for the treatment of dyspnea. J Hosp Palliat Nurs.

[14] Shimoyama N, Shimoyama M (2002). Nebulized furosemide as a novel treatment for dyspnea in terminal cancer patients. J Pain Symptom Manage.

[15] Boyden JY, Conner SR, Otolorin L (2015). Nebulized medications for the treatment of dyspnea: a literature review. J Aerosol Med Pulm Drug Deliv.

[16] Simon ST, Higginson IJ, Booth S, Harding R, Weingärtner V, Bausewein C (2016). Benzodiazepines for the relief of breathlessness in advanced malignant and non-malignant diseases in adults. Cochrane Database Syst Ref.

[17] Oxberry SF, Lawrie I (2009). Symptom control and palliative care: management of breathlessness. Br J Hosp Med (Lond).

[18] Ogle K Pain relief for terminally ill patients. Michigan State University College of Human Medicine website.

[19] Stevens RA, Ghazi SM Routes of opioid analgesic therapy in the management of cancer pain. Cancer Control.

[20] Ekström M, Nilsson F, Abernethy AA, Currow DC (2015). Effects of opioids on breathlessness and exercise capacity in chronic obstructive pulmonary disease: a systematic review. Ann Am Thorac Soc.

[21] Cohen SP, Dawson TC (2002). Nebulized morphine as a treatment for dyspnea in a child with cystic fibrosis. Pediatrics.

[22] Lang E, Jedeikin R (1998). Acute respiratory depression as a complication of nebulised morphine. Can J Anaesth.

[23] Currow DC, Mcdonald C, Oaten S (2011). Once-daily opioids for chronic dyspnea: a dose increment and pharmacovigilance study. J Pain Symptom Manage.

[24] Scarpaci LT, Tsoukleris MG, McPherson ML (2007). Assessment of hospice nurses’ technique in the use of inhalers and nebulizers. J Palliat Med.

[25] Woelk C (n.d). Practical management of common problems in palliative care. The College of Family Physicians of Canada website.

